# A capsule-dependent lytic phage for targeting multidrug-resistant and hypervirulent *Klebsiella pneumoniae*

**DOI:** 10.1128/aem.01380-25

**Published:** 2025-10-31

**Authors:** Ziyan Tian, Lin Gan, Junxia Feng, Guanhua Xue, Bing Du, Jinghua Cui, Chao Yan, Hanqing Zhao, Yanling Feng, Zheng Fan, Tongtong Fu, Ziying Xu, Zihui Yu, Yang Yang, Ke Yuehua, Xiaohu Cui, Zhijie Tang, Jing Yuan

**Affiliations:** 1 Capital Institute of Pediatrics, Chinese Academy of Medical Sciences & Peking Union Medical College, Beijing, China; 2 Department of Bacteriology, Capital Center for Children's Health, Capital Medical University, Capital Institute of Pediatrics, Beijing, China; University of Nebraska-Lincoln, Lincoln, Nebraska, USA

**Keywords:** *Klebsiella pneumoniae*, multidrug-resistant, hvKp, phage, *wcaJ*

## Abstract

As the threat of multidrug-resistant *Klebsiella pneumoniae* strains rises, the potential of phages as promising alternatives to antibiotics is increasingly being demonstrated. In this study, we isolated and characterized phiK2044, a highly specific and efficient lytic phage targeting the model strain *K. pneumoniae* NTUH-K2044. Demonstrating wide host compatibility, potent lytic activity, and robust environmental adaptability, phiK2044 exhibited exceptional efficacy against hypervirulent subtypes, including hypervirulent *K. pneumoniae* (45.2%), sequence type 23 (87.5%), and capsular K1 (92.3%). In the mouse model, phiK2044 effectively cleared bacteria without significant side effects, highlighting its therapeutic potential. Mechanistically, we identified *wcaJ*, a gene encoding a glycosyltransferase essential for capsular synthesis, as the critical determinant of the binding of phiK2044 to the host. Beyond its clinical utility, phiK2044 represents a model for studying phage ecology, host–microbe interactions, and capsule-dependent tropism. Collectively, phiK2044 represents a valuable tool against ST23/K1 *K. pneumoniae* infections, such as liver abscesses and bacteremia.

**IMPORTANCE:**

The rise of multidrug-resistant *Klebsiella pneumoniae* demands innovative therapies. This study identifies phiK2044, a lytic phage with high specificity and efficacy against hypervirulent subtypes. It safely clears infections in mice and reveals *wcaJ*-dependent capsule synthesis as the key host interaction mechanism. Beyond its therapeutic promise, phiK2044 serves as a critical tool for studying phage-host dynamics and capsule-mediated tropism, bridging clinical solutions and fundamental research in combating antimicrobial resistance.

## INTRODUCTION

*Klebsiella pneumoniae* is a Gram-negative bacterium belonging to the *Enterobacteriaceae* family and is a conditional pathogen (1). *K. pneumoniae* is usually present in the flora of the nose, throat, skin, and intestines of healthy people, and it is also commonly found in the natural environment related to humans (2). It mainly causes various diseases in older adults, children, immunocompromised individuals, and inpatients, including people with pneumonia, sepsis, pyogenic liver abscess, bacteremia, and meningitis, and infection by this microbe greatly increases the mortality rate of immunocompromised people (3). In addition, *K. pneumoniae* is the second most common cause of bloodstream infection among Gram-negative bacteria after *Escherichia coli* (3).

However, with antibiotic misuse and the resulting selective pressure on microbes, drug resistance is an increasingly serious problem among *K. pneumoniae* populations. For example, the resistance rate of *K. pneumoniae* to carbapenems in the United States is approximately 10%–15%, whereas rates of 20%–50% have been reported in China. In addition, drug-free superbugs are also frequently reported, bringing severe challenges to clinical treatment (4). According to the WHO, *K. pneumoniae* has become a global priority multidrug-resistant (MDR) bacterium (5). Therefore, there is an urgent need for new treatment methods for *K. pneumoniae* prevention and control.

*K. pneumoniae* can be classified into two distinct phenotypes based on its virulence: classical *K. pneumoniae* (cKp) and hypervirulent *K. pneumoniae* (hvKp) (6). Although cKp was more prevalent in the past and it mainly caused infections in immunocompromised individuals, leading to severe antibiotic resistance, hvKp with a hypermucoviscous phenotype has become the major clinical pathogen in recent years. In addition, hvKp strains both infect immunocompromised individuals and cause disease in healthy people. According to statistics, approximately half of hvKp infections occur in healthy young individuals (7). Due to the overuse of antibiotics and the rapid evolution of pathogens, MDR hvKp strains have been increasingly reported globally, exhibiting resistance to multiple antibiotics and high virulence. Particularly concerning are carbapenem-resistant and hypervirulent *K. pneumoniae* (CR-hvKp) strains, which cause difficult-to-treat infections in healthy individuals. Recently, outbreaks of CR-hvKp sequence type 11 (ST11) and ST15 strains were reported in Anhui, China, posing a major threat to public health (8). Therefore, the treatment of MDR hvKp, which poses a serious challenge to public health, is urgent and significant.

Phage therapy is a novel approach to treating bacterial infections. Compared with treatment using broad-spectrum antibiotics, phages have irreplaceable advantages, such as high specificity, good adaptability to the internal environment of the body, and no side effects on the host (9). Therefore, using phage therapy instead of traditional antibiotic therapy can solve the problem of bacterial resistance and achieve better therapeutic effects.

According to a report by Roach et al., phage therapy is highly effective in treating acute pneumonia caused by MDR *Pseudomonas aeruginosa* (10). In the report by Chadha et al., phages successfully replaced antibiotics in the treatment of bacterial wound infections caused by *K. pneumoniae* in mice (11). Laforêt et al. reported that phages were effective in treating urinary tract infections caused by *K. pneumoniae*, and significant *in vivo* efficacy was observed in a borer larvae model, effectively reducing larval mortality (12). In addition to infectious diseases caused by MDR bacteria, phage therapy can also effectively treat metabolic diseases caused by these pathogens. According to research by Duan et al., phages are capable of treating alcohol-induced liver disease caused by *Enterococcus faecalis* (13). Similarly, we also demonstrated that phages can treat non-alcoholic fatty liver disease caused by MDR *K. pneumoniae* (14). Moreover, no significant pathological injuries were observed in the aforementioned studies. Phage therapy holds immeasurable potential, offering unparalleled prospects for the treatment of both infectious and metabolic diseases caused by MDR pathogens.

However, phages also have a corresponding resistance mechanism. In the first step of infection, phages attach to a receptor on the bacterial surface (15). These receptor binding sites are typically protein or sugar portions on bacterial cells that are recognized by phage proteins, making the phage specific for the host, and receptor mutations are among the most common causes of resistance to phages (1, 16). To better use phage therapy, it is also important to analyze and clarify the mechanism of phage resistance.

NTUH-K2044 is a *K. pneumoniae* strain isolated from a patient with liver abscess, and it is classified as hvKp, ST23, and capsular K1, all of which have been linked to a hypervirulent phenotype of *K. pneumoniae*. Numerous studies have demonstrated the high virulence of NTUH-K2044, making it a highly representative strain for research on hvKp (17). To investigate the potential efficacy of phages in the treatment of prevalent MDR hvKp infection, we isolated the specific phage phiK2044 targeted for NTUH-K2044 from hospital sewage. We initially identified the biological and genomic characteristics, along with the host range of phiK2044, to validate its potential for tolerance in application environments and its scope for clinical application. Furthermore, we established a mouse model of bacteremia to evaluate the therapeutic effect of phiK2044. Meanwhile, we constructed a recombinant strain to explore the mechanism by which receptor mutation confers resistance to phages. These findings established a genuine and dependable foundation for the clinical application of phages in treating infectious diseases, while also providing a robust theoretical framework for addressing the challenge of phage resistance.

## MATERIALS AND METHODS

### Strains, cells, medium, and culture conditions

The strain NTUH-K2044 was purchased from the China General Microbiological Culture Collection Center. NTUH-K2044 was cultivated in Luria–Bertani (LB) liquid medium maintained at 37°C. LB liquid medium was prepared by dissolving 25 g of LB powder in 1 L of ultra-pure water. Lung adenocarcinoma (A549), hepatocellular carcinoma (HepG2), and colorectal adenocarcinoma (HT29) cells were acquired from the National Infrastructure of Cell Line Resource platform. These cells were grown in DMEM medium as the base, with the complete growth medium consisting of DMEM supplemented with 10% fetal bovine serum. The cells were cultured at 37°C in an environment containing 5% CO_2_.

### Analysis of antimicrobial resistance genes in bacterial host isolates

ResFinder software (version 4.0) was used to predict the antibiotic resistance gene profiles. This database encompasses resistance genes related to a wide range of antibiotics, including aminoglycosides, beta-lactams, fusidic acids, fluoroquinolones, fosfomycin, colistin, macrolides, glycopeptides, nitroimidazoles, lincosamide–streptogramin B complex, oxazolidinones, phenols, pseudomonic acids, tetracyclines, rifampicin, sulfonamides, and trimethoprim, as well as multidrug resistance genes. All these resistance genes are cataloged on the Epidemiology Center’s genome website (https://www.genomicepidemiology.org/).

### Isolation and purification of phages

Using the *K. pneumoniae* isolate NTUH-K2044 as the host strain, the phage was isolated from untreated hospital sewage. Initially, the sewage was centrifuged at 10,000 rpm for 5 min to eliminate large impurities. Subsequently, the supernatant was passed through a 0.22 µm syringe filter to remove bacterial contaminants. Next, 200 µL of the filtered supernatant was combined with 100 µL of the host strain in log phase and introduced into 5 mL of LB broth. After shaking the mixture for 2 h, 100 µL of the clear culture (free of visible bacteria) was mixed with 50 µL of the host strain in log phase and incubated overnight on a double-agar plate. The resulting single phage plaque was then purified three consecutive times using the double-agar plate technique.

### Host range identification

The testing strains consisted of 81 clinical *K. pneumoniae* isolates sourced from patients with pulmonary infections, urinary tract infections, or liver abscesses across nine provinces in China. For the experiment, the host range of the bacteriophage phiK2044 was determined using a combination of double-layer agar plate assay and liquid lysis activity assay. The double-layer agar plate assay: applied 10 µL phage suspension (1 × 10^8^ PFU/mL) to each bacterial lawn and incubated at 37°C overnight. Clear plaques indicated susceptible hosts. The liquid lysis activity assay: combined 200 µL phage lysate with 100 µL bacterial culture in 5 mL LB broth, and incubated with shaking for 2 h. Culture broth clarification indicated susceptible hosts.

### Transmission electron microscopy

The enriched phages were resuspended with 200 µL of PBS and then negatively stained with 2% (wt/vol) uranium acetate (pH 7.0) and observed by transmission electron microscopy (TEM) at 80 kV.

### Optimal multiplicity of infection testing

Phage particles and logarithmic-phase host bacteria were introduced into LB liquid medium at optimal multiplicities of infection (MOIs) of 0.0001, 0.001, 0.01, 0.1, 1, 10, and 100. After shaking the cultures at 200 rpm for 2 h, the optimal MOI treatment group exhibited the highest number of bacteriophages on the double-layer plate.

### One-step growth curve

The host strain was infected with phages under optimal MOI conditions, and the number of phages was measured every 10 min during the 150 min period of infection. In the one-step growth curve assay, the latent and burst periods of phage growth were determined, and the burst size of the phage was calculated using the ratio of the final number of phages to the initial number of bacteria.

### Lysis activity of phiK2044

NTUH-K2044 at a concentration of 1 × 10^9^ colony-forming units (CFU)/mL was mixed with 100 µL of phage cultures with MOIs of 1 × 10^−8^, 1 × 10^−7^, 1 × 10^−6^, 1 × 10^−5^, 1 × 10^−4^, 1 × 10^−3^, 1 × 10^−2^, 1 × 10^−1^, 1 and 10. Then, the optical density at 600 nm (OD_600_) was measured using a microplate reader. The plate was subjected to shaking culture at 37°C and 200 rpm for 7 h, and OD_600_ was measured every hour.

### Thermal stability and pH stability

Aliquots of the phage cultures with a volume of 1 mL and a concentration of 1 × 10^9^ PFU/mL were placed in a metal bath at 4°C, 10°C, 20°C, 30°C, 40°C, 50°C, 60°C, 70°C, or 80°C for 1 h. Subsequently, the titer of the phage was determined by the double-layer agar plate method. LB liquid broth with a pH ranging from 1 to 14 was prepared using 1 M hydrochloric acid and 1 M sodium hydroxide. Then, 500 µL of the phage cultures at a concentration of 1 × 10^9^ PFU/mL were mixed with 500 µL of the LB broth at different pH values. The mixtures were incubated at 37°C for 1 h. Subsequently, the titer of the phage was determined by the double-layer agar plate method.

### Cytotoxicity testing

A549, HepG2, and HT29 cells were placed in 96-well plates at a density of 4 × 10^4^ cells per well and incubated overnight. The cells in the high, medium, and low concentration groups were cultured with 100 µL of phage cultures at concentrations of 1 × 10^5^, 1 × 10^7^, and 1 × 10^9^ PFU/mL for 3 h. The formula for calculating cytotoxicity was as follows: cytotoxicity = (lactate dehydrogenase [LDH] release in the treatment group − LDH release in the negative control group)/(LDH release in the positive control group − LDH release in the negative control group).

### Host mutagenesis assay

The culture was mixed at an MOI of 0.001, and the resulting mixture was incubated in LB broth at 37°C with shaking at 200 rpm for 24 h. Subsequently, the mixture was overlaid onto a double-layer agar plate and incubated at 37°C for 12 h to permit the formation and selection of colonies. Next, the phage-resistant mutant isolates were analyzed by whole-genome sequencing (18).

### Construction of knockout strain

To construct the *wcaJ* knockout plasmid, the upstream (Primers: F- TTGCGGCCGCCCTTAATTGGAACA, R- GCAAACTTTAAGCGCGATGCATTAGC) and downstream (Primers: F- GCTAATGCATCGCGCTTAAAGTTTGC, R- TTGCGGCCGCACCATCTTCACATAAT) homologous arm fragments were amplified from the wild-type strain NTUH-K2044. The obtained overlap fragment was then cloned into the pKO3 plasmid which was digested with the restriction enzyme *Not*I. The pKO3 derivative pKO3-*wcaJ* was electroporated into DH5α for modification and transformed into NTUH-K2044 for allelic replacement as previously described (19). PCR (Primers: pKO3_F-AATAAGCGGATGAATGGCAG, pKO3_R-TCCCTCACTTTCTGGCTGG, *wcaJ*_F-GGCTCGTTACCTGCTGGATT, *wcaJ*_R-CGGAATATAACGGGTCCGGG) and sequencing were used to verify the mutation (Table S1).

### *K. pneumoniae* infection and phage therapy in mice

The animal models used in this study were 7-week-old female BALB/c mice (Charles River Laboratories, Wilmington, DE, USA). All experimental procedures involving these mouse models were approved by the Medical Ethics Committee of the Capital Institute of Pediatrics (DWLL2024022).

First, the mice were injected intraperitoneally with bacterial solutions of different concentrations (1 × 10^2^, 1 × 10^3^, 1 × 10^4^, 1 × 10^5^, and 1 × 10^6^ CFU). The survival of the experimental mice was then regularly monitored for 10 days. Subsequently, the median lethal dose (LD50) was calculated.

Then, the bacteremia model was established. The mice were injected intraperitoneally with a bacterial solution at a dose corresponding to 0.5 LD50 (20). After the bacteremia model was established, the mice were gavaged with different doses of phages (1 × 10^3^, 1 × 10^4^, 1 × 10^5^, 1 × 10^6^, and 1 × 10^7^ PFU) (21) at 1 h post-infection. At regular intervals (6, 24, 48, 72, and 96 h), the livers, lungs, and blood of the mice were periodically collected to determine the phage and bacterial loads. The blood samples were additionally used for cytokine measurement (IL-6/IL-1β/TNF-α), whereas the liver and lung tissues were subjected to hematoxylin–eosin (HE) staining.

### Statistical analysis

The data were presented as the mean ± standard deviation. The analysis was performed using two-way analysis of variance (ANOVA) using SPSS 20.0 (IBM Corp., Armonk, NY, USA). Statistical significance was indicated by *P* < 0.05 (*) or <0.01 (**) or <0.001 (***) or <0.0001 (****).

## RESULTS

### Phage phiK2044 targets MDR hvKp isolates

**Fig 1:**
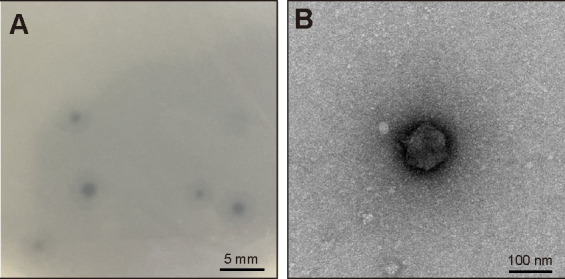
Morphology of phiK2044. (**A**) Phage plaques of phiK2044 visualized on a double-agar plate. This image shows phiK2044’s clear and round plaques, demonstrating its lytic activity. (**B**) Transmission electron microscope image presenting the morphology of phiK2044. This image shows phiK2044 is a typical *Podoviridae* family virus, characterized by a short tail and an icosahedral head with a diameter of approximately 100 nm.

MDR hvKp NTUH-K2044 is a representative typical MDR hvKp strain isolated from a patient with pyogenic liver abscess in Taiwan. Thus, we selected this isolate as the host bacterium for phage isolation. A lytic phage was isolated from hospital sewage in Beijing, China, and we named this phage phiK2044 (GenBank accession number PQ678706, CGMCC 46178, https://www.cgmcc.net/). As presented in Fig. 1A, phiK2044 formed transparent and round patches with a transparent patch in the center on the agar plate. TEM illustrated that phage phiK2044 was a typical *Podoviridae* family virus with a short tail, possessing a 100 nm diameter icosahedral head and a 20 nm non-contractile tail (Fig. 1B).

In addition to lysing NTUH-K2044, phage phiK2044 can also lyse other parts of *K. pneumoniae* isolates. We tested the host range of phiK2044 using 81 clinical *K. pneumoniae* isolates comprising various subtypes, and 17 isolates were verified to be lysed by phiK2044 (Table S2). As presented in Table 1, phiK2044 was capable of lysing most isolates of hypervirulent subtypes, such as hvKp (14/31, 45.2%), ST23 (7/8, 87.5%), and capsular K1 (12/13, 92.3%). Moreover, these 17 isolates carried multiple antimicrobial resistance genes, including 51 resistance genes, such as *acrD*, *Klebsiella_pneumoniae_OmpK37*, and *SHV-108* (Table S3). These resistance genes belong to 13 categories of antibiotics: aminoglycoside, aminocoumarin, beta-lactam, fosfomycin, fluoroquinolone, macrolides-lincosamides-streptogramin B, nitroimidazole, phosphonic acid, rifampicin, sulfonamide, tetracycline, trimethoprim, and multidrug. This suggests an urgent clinical need for novel therapies to replace antibiotics against hvKp.

**TABLE 1:** The host range profile of phage phiK2044^*a*^

Isolates	hvKp	ST	Capsular type	Accession number
QD70	hvKp	ST86	K2	JAJOXR000000000
Q22	hvKp	ST23	K1	JAJOXE000000000
14896	hvKp	ST700	K1	GCA_046467855.1
QD52	hvKp	ST367	K1	JAJOXL000000000
10430	hvKp	ST23	K1	GCA_046468855.1
DT189	hvKp	ST23	K1	JAJOVT000000000
QD51	hvKp	ST700	K1	JAJOXK000000000
QD53	hvKp	ST700	K1	JAJOXM000000000
11796	cKp	ST23	K1	GCA_046468595.1
SY7	hvKp	ST23	K1	JAJOYK000000000
12333	hvKp	ST23	K1	GCA_046468455.1
C26	cKp	ST45	K24	JAJOTY000000000
C46	hvKp	ST15	K19	JAJOUS000000000
10836	hvKp	ST86	K2	GCA_046468775.1
DT220	cKp	ST3132	K142	JAJOVU000000000
16205 NTUH-K2044	hvKp hvKp	ST367 ST23	K1 K1	GCA_046467435.1 GCA_000009885.1

^*a*^ HvKp, hypervirulent *K. pneumoniae*; cKp, classical *K. pneumoniae*; ST, sequence type. The testing strains consisted of 81 clinical *K. pneumoniae* isolates sourced from nine provinces in China.

### Phage phiK2044 exhibits effective lytic ability and environmental stress tolerance

**Fig 2:**
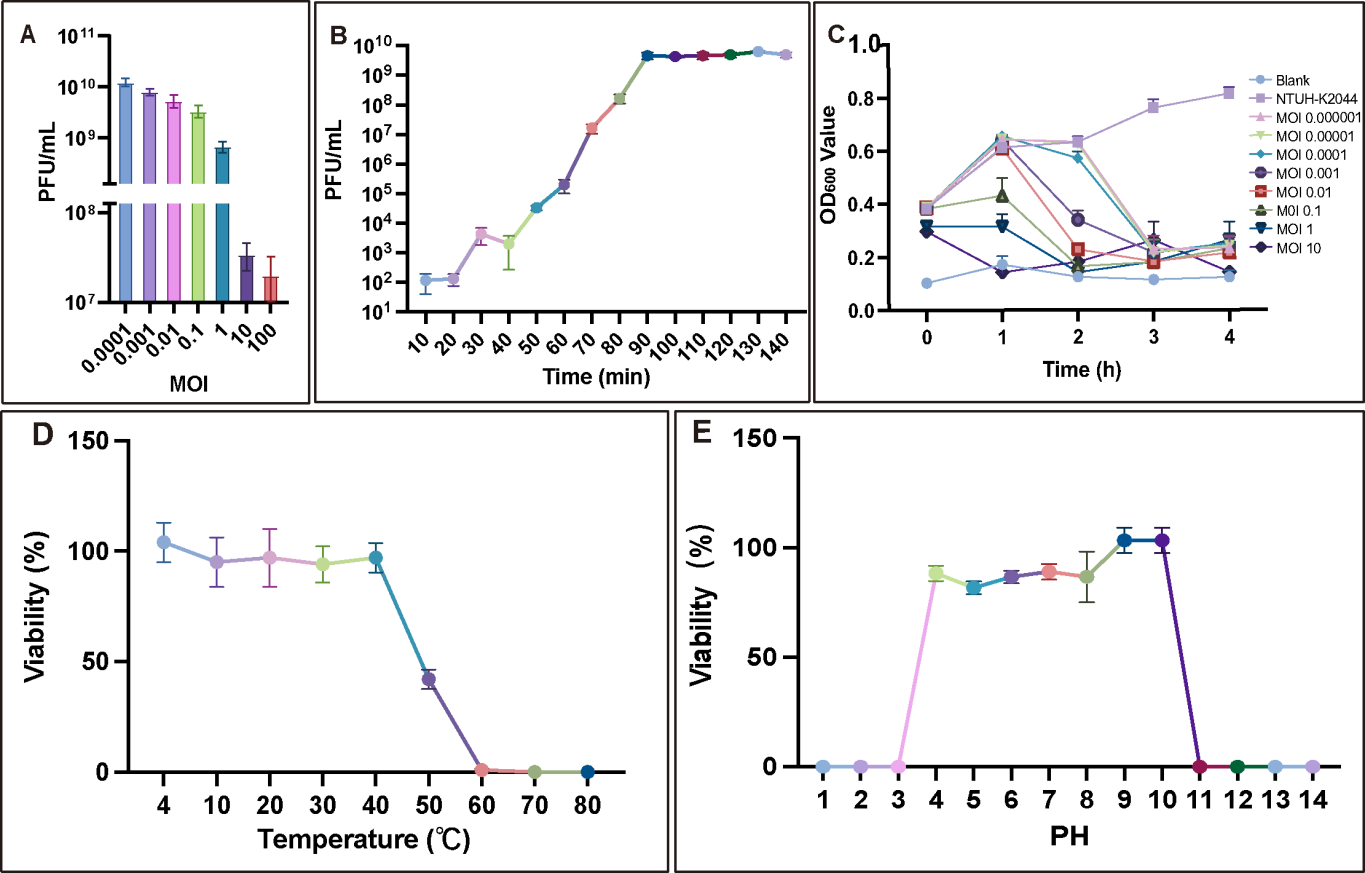
Life cycle parameters of the phage phiK2044. (**A**) Identification of the optimal MOI. (**B**) In one-step growth curves, the host-to-phage ratio was set at the optimal MOI. (**C**) Kill curves, showing suppression of host bacterial growth across a range of MOIs. (**D and E**) Stability analysis of the phage under various temperatures and pH values. Values are presented as the mean ± standard deviation. Values are presented as the mean ± standard deviation.

To identify the lytic capacity of phiK2044, the MOI, one-step growth curve, and host strain growth curve were determined. The optimal MOI of phiK2044 was 0.0001 (Fig. 2A), indicating that phiK2044 can effectively infect and lyse hvKp isolates even at an extremely low phage-to-bacteria ratio. The subsequent experiments were performed at an MOI of 0.0001. As illustrated by the one-step growth curve (Fig. 2B), the latent period of phiK2044 was approximately 20 min, the burst time was 90 min, and the average burst size was 70 PFU/cell. In terms of inhibiting the proliferation of host bacteria, phage phiK2044 could completely inhibit the growth of NTUH-K2044 at an MOI of 0.0001–10 (Fig. 2C). These results suggest that phiK2044 is a potential candidate for MDR *K. pneumoniae* infection treatment.

In addition, phiK2044 maintained high biological activity across a wide range of temperatures (4°C–50°C, Fig. 2D) and pH conditions (pH 4–10, Fig. 2E), demonstrating its potential for application in diverse environments. Thermal stability and pH stability facilitate *in vivo* phage treatment and *in vitro* phage storage and transport.

### Phage phiK2044 without genes related to antibiotic resistance, virulence, and integration

Phage phiK2044 was a circular double-stranded DNA of 43,104 bp in length with 54.02% GC content. Comparative analysis revealed that phiK2044 contains 48 putative open-reading frames, encoding proteins, such as DNA replication proteins, DNA packaging proteins, structural proteins, holin, and endolysin (Table S4). The proteins associated with host bacterial lysis are holin and endolysin, which primarily function to disrupt the host cell membrane and lyse the host bacterium, respectively. The endolysin protein in this study lacks transmembrane domains. Notably, no genes associated with antibiotic resistance, virulence, or integration were identified, indicating the potential therapeutic applicability of phiK2044 and reducing risks of lysogenic conversion or toxicity, supporting therapeutic safety.

**Fig 3:**
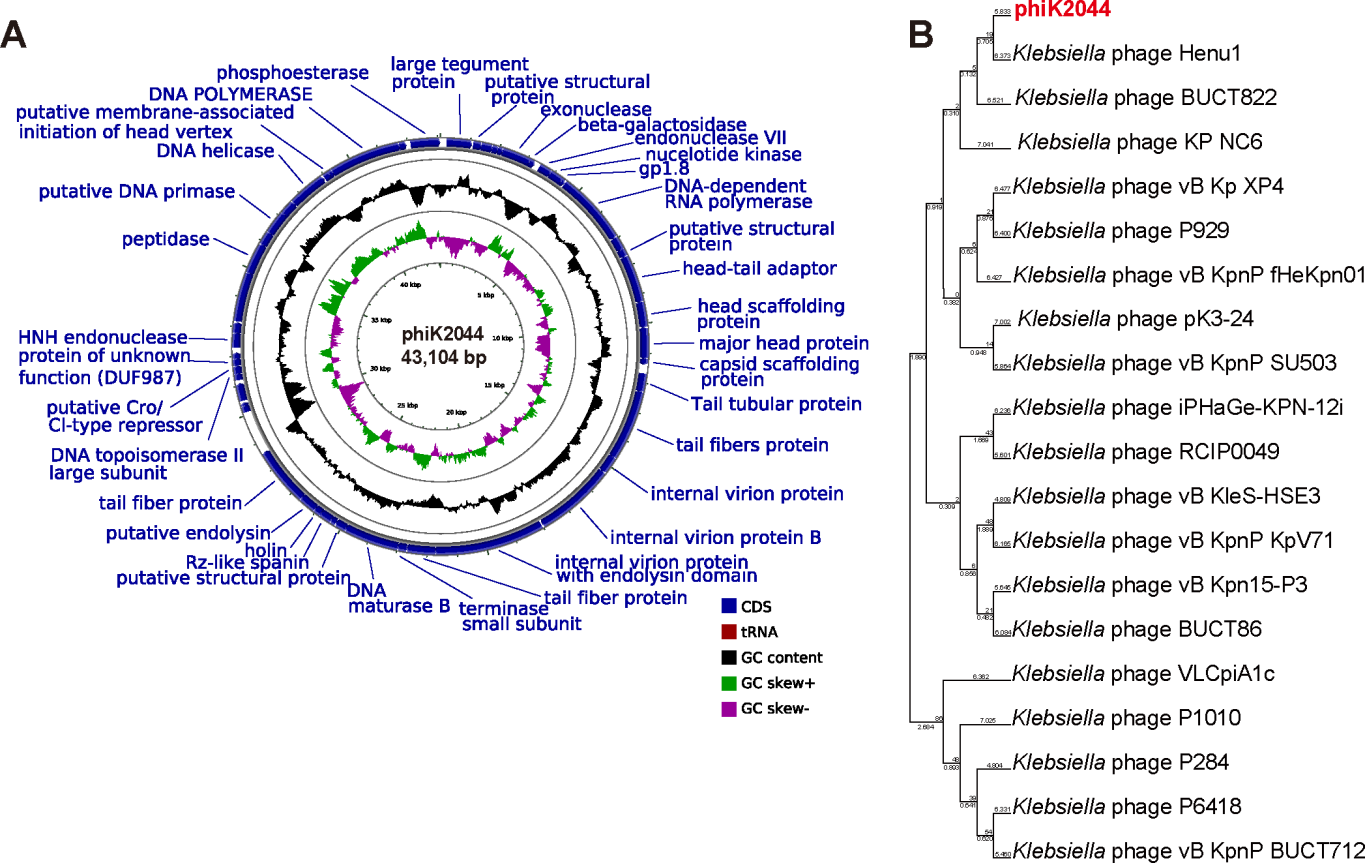
Genomic information of phage phiK2044. (**A**) Genomic map illustrating the structure of phiK2044. This genomic map provides a detailed overview of the phage’s genetic organization, including the locations of open reading frames encoding various proteins, such as DNA replication proteins, DNA packaging proteins, structural proteins, holin, and endolysin. (**B**) A phylogenetic tree was constructed using the maximum likelihood method based on the complete genomes of phages. It places phiK2044 within the Podoviridae family and reveals its genetic divergence from other known phages, supporting its unique taxonomic status and evolutionary history.

The phylogenetic tree of phages was constructed using complete genome sequences. As illustrated in Fig. 3A, phiK2044 was clustered into the *Podoviridae* family, a taxonomic designation consistent with its morphological characteristics. Furthermore, phylogenetic analysis revealed a clear divergence between phiK2044 and other known phages (Fig. 3B), as supported by significant genetic distinctions.

### Significantly low or virtually absent cytotoxicity of phage phiK2044

**Fig 4:**
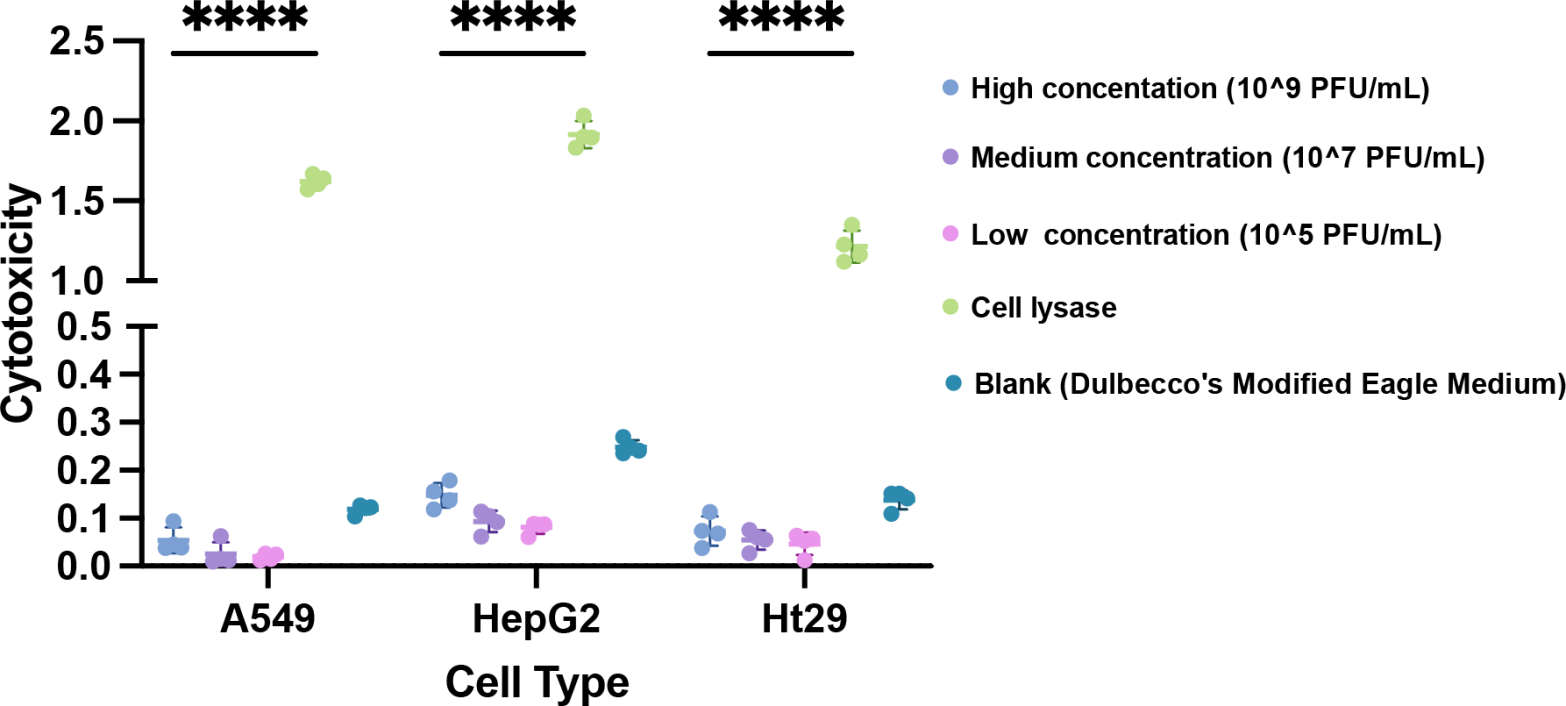
Cytotoxicity assessment of phage phiK2044 in A549, HepG2, and HT29 cells. The cytotoxicity of phiK2044 in cells was evaluated after 3 h of incubation by measuring LDH levels in the culture supernatant. High concentration, 10^9^ PFU/mL. Medium concentration, 10^7^ PFU/mL. Low concentration, 10^5^ PFU/mL. Blank, Dulbecco’s Modified Eagle Medium. Values are presented as the mean ± standard deviation. Statistical significance was determined using two-way ANOVA. ****, *P* < 0.0001.

Bacteriophages possess the ability to enter the organism and disseminate through the bloodstream to reach various target organs. Given that the lungs, liver, and intestines are the most common target sites of *K. pneumoniae* infection, this study evaluated the cytotoxic effects of phiK2044 on A549, HepG2, and HT29 cells to assess its potential toxic side effects. Cytotoxicity was systematically examined by exposing these cell lines to three concentrations (high, 1 × 10^9^ PFU/mL; medium, 1 × 10^7^ PFU/mL; low, 1 × 10^5^ PFU/mL) of phiK2044. As presented in Fig. 4, the cytotoxicity of phiK2044 was significantly lower than that of the control (lysate: the positive control group) at all three concentrations in all three cell lines (two-way ANOVA, *P* < 0.0001). Moreover, there was no significant difference in cytotoxicity between the phiK2044 and blank groups (two-way ANOVA, *P* > 0.05). The results provide insights into the safety profile of the phage for potential therapeutic applications.

### Phage phiK2044 binds to host bacteria via *wcaJ*-regulated capsules

**Fig 5:**
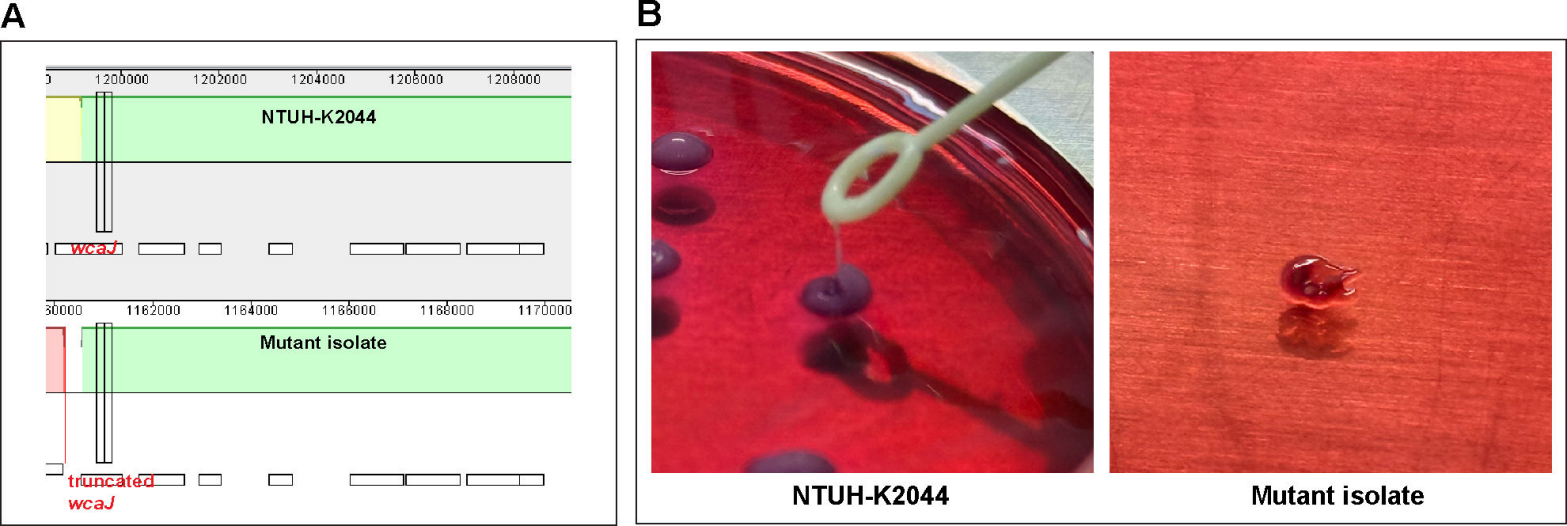
Analysis of phage–host interaction. (**A**) The genomic difference between the wild-type and mutant strain. (**B**) String test. For the string test, the *K. pneumoniae* colony on blood agar was touched with a loop and lifted vertically to check for string formation.

To identify the receptor through which phiK2044 binds to its host strain NTUH-K2044, we first conducted a host bacterium mutagenesis induction experiment, which led to the isolation of two mutant isolates that exhibited tolerance to phiK2044. Whole-genome sequencing revealed that the mutant isolate had a truncation of the *wcaJ* gene (Fig. 5A). The WCAJ protein, encoded by *wcaJ*, is an essential glycosyltransferase required for capsular synthesis, and it is located intracellularly. Its inactivation results in the termination of capsular synthesis. Furthermore, the string test and capsular typing identification demonstrated that the mutant isolates changed from string test-positive to string test-negative, and their capsular type shifted from K1 to untyped capsular type, indicating alteration or loss of the capsular structure (Fig. 5B). Integrating genomic data with phenotypic experimental analyses, it was suggested that phage phiK2044 might specifically bind to the host bacterium via the capsule through a mechanism regulated by WCAJ. To further validate this hypothesis, we subsequently constructed a knockout strain of *wcaJ*. Consistent with the previous results, *wcaJ* deletion rendered the strain resistant to phage phiK2044, resulting in a negative string test and an untyped capsular type. However, upon complementation with *wcaJ*, all phenotypic changes were restored, including restored sensitivity to phiK2044. These results demonstrate that phiK2044 binds to its host bacterium NTUH-K2044 by specially targeting the capsule through a process regulated by *wcaJ*.

### Phage phiK2044 targeting *K. pneumoniae* effectively alleviated inflammation *in vivo*

To further assess the safety, pharmacokinetics, and therapeutic efficacy of phiK2044 *in vivo*, we established a *K. pneumoniae* infection model in BALB/c mice via intraperitoneal injection. We first determined the LD50 of NTUH-K2044 in BALB/c mice via intraperitoneal administration to 316 CFU (20). Given that the uncertainty variability of phage activity *in vivo* and the optimal MOI of the phage were extremely low (316 × 0.0001 < 1 PFU), we conducted preliminary evaluations of therapeutic efficacy by testing five distinct phage doses (1 × 10^3^, 1 × 10^4^, 1 × 10^5^, 1 × 10^6^, and 1 × 10^7^ PFU) (21). The results indicated that phage doses ranging from 1 × 10^4^ to 1 × 10^7^ PFU significantly reduced or eliminated bacterial loads in mice. Thus, the subsequent murine infection models were established via intraperitoneal injections of 0.5 LD50 (158 CFU) of NTUH-K2044, and a phage dose of 1 × 10^4^ PFU was selected for subsequent therapeutic efficacy testing.

**Fig 6:**
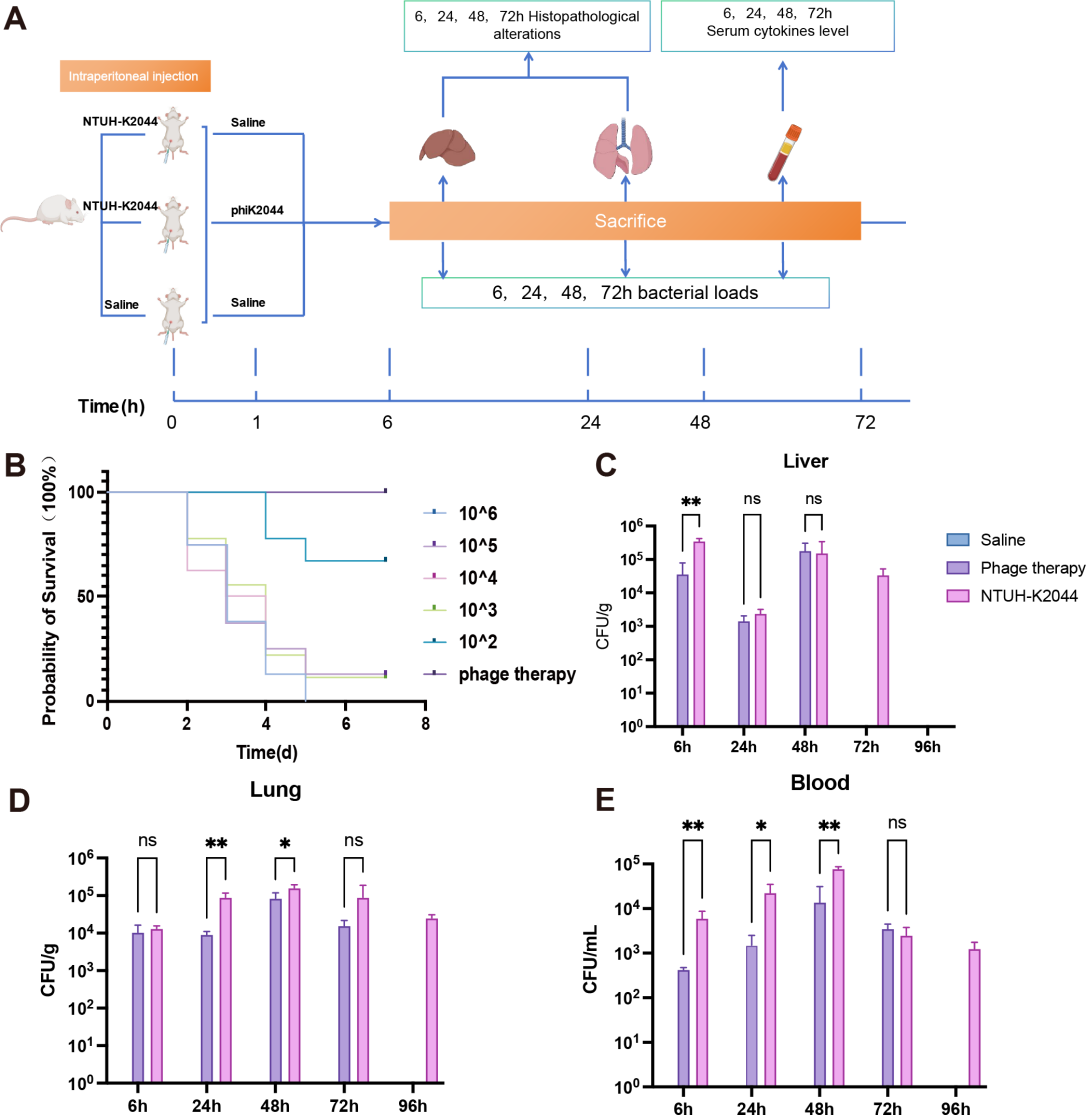
Phage therapy in mouse models. (**A**) Workflow for murine bacteremia model and phage therapy. BALB/c mice were infected with 0.5 LD50 NTUH-K2044 (158 CFU). At 1 h post-infection, phiK2044 (1 × 10^4^ PFU) was administered orally. Tissues (liver, lung, blood) were collected at 6, 24, 48, 72, and 96 h for bacterial and phage quantification and cytokine analysis. (**B**) Survival rates of mice infected with graded bacterial doses (10² to 10⁵ CFU) and subsequent phage therapy. Bacterial loads in the liver (**C**), lung (**D**), and blood (**E**) were monitored until *K. pneumoniae* clones were eliminated in the phage-treated group. Missing bars represent time points where *K. pneumoniae* was not detected. Values are presented as the mean ± standard deviation. Statistical significance was determined by two-way ANOVA. *, *P* < 0.05; **, *P* < 0.01.

This model was used to evaluate the therapeutic effect of phiK2044 *in vivo* (Fig. 6A). As illustrated in Fig. 6B, the administration of phiK2044 resulted in an immediate restoration of the survival rate to 100% in treated mice. Analysis of bacterial loads within the treated mice also demonstrated that the phage effectively eradicated the bacteria (Fig. 6C through E). Specifically, in the liver, phiK2044 completely cleared the bacteria within 72 h post-treatment, whereas the untreated group maintained a significant bacterial load (4.3 × 10^4^ CFU/g). Similarly, in the lungs and blood, the bacterial load in the phage-treated group remained consistently lower than that in the untreated group, culminating in complete eradication of the host bacteria by 96 h.

**Fig 7:**
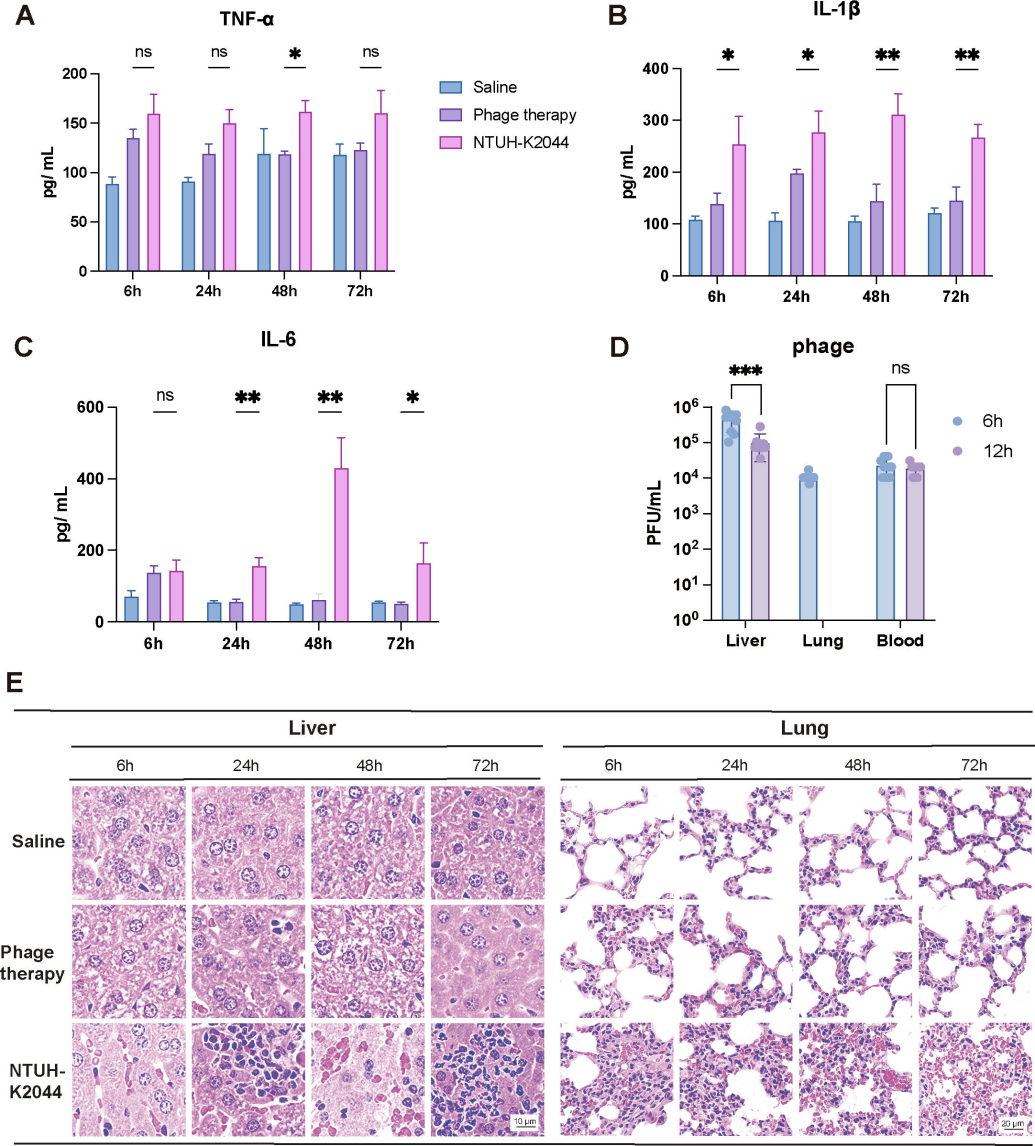
phiK2044 alleviates the inflammatory level in the mouse. (**A–C**) Concentrations of pro-inflammatory cytokines IL-6, IL-1β, and TNF-α in the blood of mice. (**D**) Phage loads in the liver, lung, and blood of mice. (**E**) Hematoxylin–eosin staining of murine liver and lung tissues. Values are presented as the mean ± standard deviation. Statistical significance was determined by two-way ANOVA. *, *P* < 0.05; **, *P* < 0.01; ***, *P* < 0.001.

Furthermore, cytokine (IL-6/IL-1β/TNF-α) concentration analysis (Fig. 7A through C) and histopathological examination via HE staining (Fig. 7E) also indicated that phage treatment significantly reduced inflammatory cytokine levels and mitigated pathological damage. For instance, HE-stained sections of the liver revealed notable inflammatory damage, including liver cell degeneration and necrosis, accompanied by substantial neutrophil aggregation. In the lung sections, pathological findings, such as hemorrhage, widened alveolar septa, collapsed alveoli, and inflammatory cell infiltration, were observed. However, these pathological alterations were completely resolved within 48 h post-treatment.

Additionally, we quantified the phage loads in various tissues. As depicted in Fig. 7D, phiK2044 successfully reached the liver, lungs, and blood of mice, achieving a higher bacteriophage load than the initial therapeutic dose, suggesting *in vivo* replication of the phage. However, phage detection was only possible at 6 and 12 h post-administration, with no detectable levels thereafter, potentially because of limitations in the sensitivity of our detection methodology or other underlying factors.

## DISCUSSION

*K. pneumoniae* is an important pathogen that can cause both nosocomial and community-acquired infections. *K. pneumoniae* typically invades the human body through the respiratory, urinary, and digestive tracts (22). In some cases, medical procedures, such as skin trauma, surgery, and catheter insertion, can also provide direct access for *K. pneumoniae* entry into the bloodstream (23). Following bloodstream entry, the microbe can spread to various sites (such as the lungs, liver, urogenital tract, and gastrointestinal tract), where subsequent colonization and proliferation lead to localized infections (24). As a clinically significant strain, NTUH-K2044, classified as hvKp of ST23 with a K1 capsular serotype, represents a crucial prototype of hypervirulent *K. pneumoniae*. In-depth investigation using NTUH-K2044 as a model organism can provide critical insights for developing novel therapeutic strategies against hypervirulent and MDR *K. pneumoniae*.

The escalating threat of MDR *K. pneumoniae* has exposed critical gaps in conventional antimicrobial strategies, and phage therapy has emerged as a promising alternative, as evidenced by previous studies (21, 25). However, comprehensive clinical validation remains necessary to establish its therapeutic efficacy, and current research priorities should focus on characterizing phages and elucidating their mechanisms to facilitate subsequent translational research and clinical implementation. In this study, we isolated and characterized the highly lytic phage phiK2044, which exerted potent activity against MDR hvKp isolates, including NTUH-K2044. Research on phiK2044 both introduces a novel therapeutic candidate and illuminates fundamental aspects of phage–bacterium interactions that could reshape approaches to combating antibiotic resistance.

Based on the comprehensive data presented in this study, the interaction between bacteriophage phiK2044 and *K. pneumoniae* is capsule-dependent, with the bacterial capsule synthesis gene *wcaJ* confirmed as the key determinant. These results raise intriguing questions about the co-evolutionary dynamics between phages and *K. pneumoniae*. Although capsular polysaccharides (CPSs) are well known for their role in shielding bacteria from immune clearance, they paradoxically serve as primary receptors for many phages—a duality that underscores the complexity of phage–host interactions (18). In this study, we observed that *wcaJ* inactivation eliminated phiK2044 infectivity, consistent with studies on *Klebsiella* phages targeting K1 and K2 capsules (21, 26). This divergence suggests that CPS-targeting phages, such as phiK2044, can impose unique selective pressures on hvKp populations, potentially driving adaptations, such as capsule switching or hypermucoviscosity, similar to those observed in *Streptococcus pneumoniae* under phage predation (27). Importantly, our complementation experiments revealed that restoring *wcaJ* expression reestablished susceptibility to phiK2044, indicating that capsule loss comes at a fitness cost (28, 29). This creates an evolutionary “trap” that limits bacterial escape routes, highlighting the dual role of CPSs as both virulence and vulnerability factors of phages. This molecular specificity accounts for the phage’s notable efficacy against hypervirulent K1/ST23 strains while naturally restricting its host range to encapsulated variants, thereby minimizing off-target effects. These findings reveal a critical vulnerability in hvKp that can be therapeutically exploited. Collectively, this mechanism establishes phiK2044 as a precisely targeted therapeutic against multidrug-resistant hvKp and simultaneously offers a valuable model system for investigating phage-capsule coevolution dynamics.

In addition, genome analysis identified that phage phiK2044 carries holin and endolysin genes. The holin-endolysin system represents the most classical phage lysis mechanism. After entering the host bacterium, holin accumulates in the cytoplasm and forms large oligomers that target the cell membrane, creating pores in the membrane. This enables endolysin to degrade peptidoglycan, ultimately leading to host bacterial lysis and release of progeny phages. However, recent studies have shown that some phages encode an endolysin containing transmembrane domains. This type of endolysin can function independently of holin by anchoring itself to the cell membrane to perform signal sensing and release functions, subsequently cleaving peptidoglycan (30, 31). In this study, the endolysin protein of phiK2044 lacks transmembrane domains and coexists with a holin protein. Therefore, we suggest that phiK2044 likely lyses host bacteria through the classical holin-endolysin pathway, though the specific mechanism requires further experimental validation.

Phage phiK2044 was capable of lysing diverse MDR hvKp subtypes (such as ST23 and K1 isolates), likely because of conserved capsular epitopes. Structure similarities in CPSs across *K. pneumoniae* isolates (such as shared glycosylation patterns in K1 or K2 serotypes) might enable cross-reactive phage binding, as suggested for *E. coli* phages targeting K1-like capsules (32, 33). In addition, the absence of integrases, antibiotic genes, or virulence genes in the phiK2044 genome further distinguishes it from partial phages that contribute to horizontal gene transfer in *Klebsiella* (e.g., phage-mediated dissemination of *rmtB* aminoglycoside resistance) (19, 34). This “clean” genomic architecture of phiK2044 both enhances therapeutic safety and holds potential for engineering phages with minimized ecological risks.

It is noteworthy that a single dose of phage therapy demonstrated remarkable therapeutic efficacy *in vivo*, as characterized by a 100% survival rate and rapid bacterial eradication. This phenomenon might not solely be attributable to the intrinsic action of phages; rather, the innate immune response likely plays a synergistic role. Specifically, the lytic activity induced by phages might expose bacterial antigens, thereby enhancing macrophage-mediated clearance mechanisms. In the subsequent detection of phage titers, phiK2044 was only detected within the first 12 h after administration. This might be related to rapid phage–bacterium co-elimination via immune mechanisms (such as complement-mediated opsonization). Of course, it is also possible that this is merely attributable to the sensitivity limitations of the detection method.

While our study focused on cytokine profiling during the peak inflammatory phase (24–72 h post-infection), we recognize that extended temporal analysis could provide valuable insights into the complete dynamics of immune modulation during phage therapy. Moreover, although current preclinical models support the translational potential of phage therapy, further clinical validation remains essential. Future studies should focus on optimizing dosing regimens and evaluating therapeutic efficacy in more complex infection models to facilitate clinical translation.

The core difference between phage phiK2044 therapy and traditional antibiotics lies in the following aspects: compared to broad-spectrum antibiotics, phiK2044 can overcome multiple resistance mechanisms, selectively lysing highly virulent K1/ST23 *K. pneumoniae*. Its capsule-dependent activity (regulated by *wcaJ*) avoids damage to the commensal microbiota, thereby significantly reducing the risk of secondary infections caused by ecological imbalance. In terms of pharmacokinetics, a single oral dose of phiK2044 enables continuous bacterial clearance and self-amplification at the infection site, whereas antibiotics typically require repeated high-dose administration, which often leads to recurrence. Regarding resistance development, resistance to phiK2044 requires loss of capsule synthesis, which attenuates virulence, whereas antibiotic resistance can spread stably via plasmids without reducing pathogenicity. Clinically, phiK2044 offers major advantages for immunocompromised patients: its precise lytic activity preserves the commensal flora, and its self-replicating nature at the site of infection overcomes the pharmacokinetic decay typical of antibiotics, enabling sustained antibacterial activity without the need for dose escalation.

These observational findings highlight the need for a new model that integrates phage dynamics, host immunity, and bacterial ecology, namely “phage pharmacology ecology,” for further in-depth research.

### Conclusions

In general, phage phiK2044 transcends its role as a therapeutic candidate, providing insights into fundamental problems in phage ecology, bacterial evolution, and host–microbe interactions. Its unique traits, including capsule-dependent tropism, genomic minimalism, and immune-compatible efficacy, challenge existing paradigms in phage therapy development. Further work should prioritize two areas, namely the mechanism of capsule-phage coevolution and the ecological monitoring frameworks to track phage-driven population shifts in *K. pneumoniae*.

## Data Availability

The complete genome sequence of phage phiK2044 has been deposited in NCBI under accession number PQ678706 and in the China National Center for Bioinformation database under accession number C_AA122191.1. The phage data have been deposited at the China General Microbiological Culture Collection Center under CGMCC 46178 and at the Capital Institute of Pediatrics, Beijing, under phiK2044-1. If viable phage strains are required for further study, please contact the corresponding author, either through the Capital Institute of Pediatrics or CGMCC. The source data of this paper are available at the Science Data Bank (https://doi.org/10.57760/sciencedb.23309).

## SUPPLEMENTAL MATERIAL

Supplemental tables aem.01380-25-s0001.xlsx Tables S1 to S4.

ASM does not own the copyrights to Supplemental Material that may be linked to, or accessed through, an article. The authors have granted ASM a non-exclusive, world-wide license to publish the Supplemental Material files. Please contact the corresponding author directly for reuse.
